# Artificial Biopolymers Derived from Transgenic Plants: Applications and Properties—A Review

**DOI:** 10.3390/ijms252413628

**Published:** 2024-12-19

**Authors:** Krystyna Latour-Paczka, Robert Luciński

**Affiliations:** Department of Plant Physiology, Faculty of Biology, Institute of Experimental Biology, Adam Mickiewicz University in Poznań, Uniwersytetu Poznańskiego 6, 61-614 Poznań, Poland; krylat@st.amu.edu.pl

**Keywords:** artificial biopolymers, artificial biopolymer applications, nanocellulose, polyhydroxyalkanoates, rubber, starch-based polymers, spider silk proteins, transgenic plants

## Abstract

Biodegradable materials are currently one of the main focuses of research and technological development. The significance of these products grows annually, particularly in the fight against climate change and environmental pollution. Utilizing artificial biopolymers offers an opportunity to shift away from petroleum-based plastics with applications spanning various sectors of the economy, from the pharmaceutical and medical industries to food packaging. This paper discusses the main groups of artificial biopolymers. It emphasizes the potential of using genetically modified plants for its production, describing the primary plant species involved in these processes and the most common genetic modifications. Additionally, the paper explores the potential applications of biobased polymers, highlighting their key advantages and disadvantages in specific context.

## 1. Introduction

In today’s world, biodegradability is becoming increasingly important as the global population continues to grow. Humanity faces the dual challenge of minimizing its environmental impact while meeting its diverse needs. Biopolymers represent a broad class of natural, biodegradable substances produced by living organisms and composed of specific biomacromolecules [1]. Due to their numerous advantages, biopolymers and their modifications have garnered significant attention in recent years, impacting industries such as biomedical, agriculture, and packaging as sustainable alternatives to petroleum-based synthetic polymers [2,3,4].

According to IUPAC guidelines, chemically synthesized copies of biopolymers are classified as synthetic biopolymers, distinguishing them from true biopolymers [1]. Polymers derived wholly or partially from plants, animals, or microorganisms are referred to as biobased polymers. The term bioplastic, often used in this context, is not recommended [1].

Another emerging source of polymers involves transgenic organisms, which are genetically modified to enhance or alter specific properties of biopolymers. These non-natural analogues, as per IUPAC recommendations, are termed artificial biopolymers. Genetic engineering methods enable the creation of such biopolymers, expanding their potential applications [1].

This review focuses on biopolymers synthesized in transgenic plants that have undergone genetic modifications to produce tailored biopolymers. To align with IUPAC nomenclature, the authors have adopted the term artificial biopolymers to describe these substances and their applications.

The world’s most abundant natural polymer is cellulose, accounting for 40–50% of the wood in gymnosperms and angiosperms [5,6]. Formed as a final result of CO_2_ assimilation and the carbon anabolic pathway, cellulose is renewable and has a low toxicity and carbon footprint, making it one of the most cost-effective and sustainable materials [6]. While cellulose is naturally synthesized by plants, transgenic approaches have been explored to alter cellulose production or introduce cellulose-related genes from other organisms to enhance polymer properties [7]. Cellulose compounds have a variety of application areas, including the biomedical field, insulating materials, packaging, and electronics [8]. Starch-based polymers are also generated in transgenic plants that can be engineered to have an increased amylose content or altered branching patterns for improved material quality. Additionally, the use of designer starch, like the amylose-only (AO) barley starch with 99% amylose content, has been explored to create a biobased polymer with comparable properties to commercial plastic materials, showcasing the potential of engineered starch in sustainable material development [9]. Furthermore, advancements in gene editing technologies such as CRISPR/Cas9 systems offer promising methods to increase amylose content in plants like potatoes, leading to improved starch characteristics and potential applications in genetic plant quality enhancement [10].

Polyhydroxyalkanoates (PHAs) are another group of artificial biopolymers whose production has gained momentum in the recent years. PHAs are originally synthesized by microorganisms in low-nutrient conditions, but their synthesis is also possible in genetically modified plants such as *A. thaliana* [11]. Valued for their biocompatibility, mechanical, and biodegradable properties, this versatile class of polymers holds great promise as a sustainable plastic alternative. Other materials include spider silk proteins, rubber, and lignin-modified polymers, all possible to produce with the use of transgenic plants and in various applications in fields spanning from pharmaceutical, agriculture, and packaging to the food industry [12].

While artificial biopolymers offer more sustainable alternatives to conventional plastics and can be applied in nearly every field, they come with their own limitations—relatively high production costs, limited scalability and recycling options, and concerns about durability, to name a few. Properties of artificial biopolymers may also not match those of some petroleum-derived plastics, which may or may not be seen as an obstacle. For instance, gas permeability of certain biomaterials might be lower than that of traditional plastics, making them unsuitable for food packaging [13].

In this review we delve into the characteristics and production of specific artificial polymers in transgenic plants. Furthermore, we explore their diverse applications and far-reaching implications across multiple sectors of the industry.

## 2. Characteristics of Selected Artificial Biopolymers

Plant-based polymers such as cellulose, starch, or rubber have been used by mankind for hundreds of years for various purposes. However, with the discovery of petroleum-derived polymers in the late 19th and early 20th centuries, their global usage decreased as the natural materials began being replaced by their more durable, heat-resistant, and easily modulable counterparts. Plastics offered a previously unimaginable combination of cost-effectiveness and versatility, alongside methods such as injection molding and blow molding, which enabled large-scale manufacturing of plastic products, displacing many previously used materials, transforming industries and everyday life. The development of specialized infrastructure for polymer manufacturing, distribution, and waste management further facilitated the widespread adaptation of petroleum-derived materials in the mid-20th century [14]. While these materials have brought numerous benefits in terms of convenience, versatility, and affordability, allowing for breakthroughs and technological advancements, it is crucial to acknowledge the problems that were created because of plastic consumption.

Currently we face challenges related to plastic pollution, resource depletion, and insufficient waste management strategies, driving interest in the development of sustainable alternatives to traditional plastics. Yet, to successfully replace them, artificial biopolymers have to meet the same requirements, and even exceed the expectations placed upon petroleum-derived materials, allowing for more efficient recycling and/or biodegradation, as well as covering the same broad spectrum of properties and applications. Generally, those properties should involve durability, strength, a sufficient shelf life for intended use, thermal stability, and optimal gas permeability. Moreover, they should meet regulatory requirements for safety, quality, and environmental impact to serve as a better alternative to traditional plastics.

Transgenic plants emerged as versatile bioreactors to produce a diverse array of artificial biopolymers, including cellulose- and starch-based polymers, polyhydroxyalkanoates (PHAs), and spider silk proteins, offering advantages such as renewable sourcing, reducing dependence on fossil fuels, and decreasing environmental impact [15,16]. These bioreactors also provide scalability, crucial for meeting industrial demands and ensuring cost-effective production on a commercial scale [17].

### 2.1. Cellulose-Based Polymers

Cellulose stands as the most abundant natural polymer, accounting for approximately 1.5 × 10^12^ tons of the yearly biomass yield. It emerges as a near limitless source of raw material to meet the escalating requirements for eco-conscious and biocompatible goods [18]. Cellulose is insoluble in water and most organic solvents, has crystalline structure, and is biodegradable [19,20]. Transgenic plants can be engineered to produce cellulose with modified properties, such as altered crystallinity or degree of polymerization, by manipulating the expression of cellulose synthase genes or introducing cellulose-modifying enzymes. In recent years, there has been a significant surge in interest surrounding cellulose biosynthesis as a sustainable alternative to traditional petroleum-based materials. This is largely due to cellulose’s distinctive attributes such as exceptional strength and stiffness, relatively low density, simple structure, versatility, and widespread availability [21]. Cellulose-based polymers produced in transgenic plants encompass a variety of materials tailored for specific applications, ranging from nanotechnology to biomedicine to sustainable packaging [22].

### 2.2. Nanocellulose (NC)

Nanocellulose, compared to its precursor cellulose, shows enhanced crystallinity, high surface area, rheological properties, alignment, and orientation [23]. Generally, the term “nanocellulose” refers to cellulose molecules which have at least one dimension in nanoscale, i.e., 1–100 nm [23]. It includes a variety of forms with different morphologies, such as fibers, crystals, and bacterial cellulose [24].

Nanocellulose-based materials are carbon-neutral, nontoxic, sustainable, and recyclable, making them suitable for a wide-range of applications. In the biomedical field, nanocellulose can be used in wound dressings, drug delivery systems, medicinal inserts, tissue engineering, and as cosmetic agents or implants [25]. In the realm of composite materials, nanocellulose is utilized for producing biodegradable plastics, aqueous coatings, and polymer adhesives [26].

At present, the commercial synthesis of nanocellulose is an entirely established technique, however, it involves harsh chemical treatments that are perpetually hazardous to humans and the environment [23,27,28]. Therefore, to apply NC on a wider scale, more sustainable and safe methods for production must be developed and scaled to sustain the industry demand.

Cellulose synthase (CS) is a crucial enzyme complex responsible for cellulose biosynthesis. The biosynthetic pathway of cellulose synthase involves the assembly of cellulose synthase complexes (CSCs) in the Golgi apparatus, their delivery to the plasma membrane for active cellulose synthesis, and their movement along cortical microtubules to define microfibril orientation. Modifying genes encoding cellulose synthase (CS) or its regulators in transgenic plants can influence the quality of nanocellulose produced [29].

Production of nanocellulose in transgenic plants involves genetic modifications that enhance the plant’s natural ability to synthesize cellulose, resulting in an increase in the nanocellulose yield. One of the methods involves targeting cellulose synthase genes (CESA), which encode enzymes that form the cellulose synthase complex responsible for the polymerization of glucose into cellulose chains. Studies on transgenic hybrid aspen trees with reduced expression of Cellulose Synthase-Interactive Protein 1 (CSI1) demonstrated alterations in wood mechanics and cellulose microfibril properties, leading to distinct nanofibril qualities with lower suspension viscosity, reduced water uptake, and higher stiffness [30,31]. These results indicate that changes in the native cellulose structure impact the final isolated cellulose nanofiber properties. Additionally, manipulating cellulose synthase enzymes, like CESA, can impact the crystallinity of cellulose, affecting its enzymatic conversion efficiency to fermentable sugars, thus potentially enhancing biofuel production from cellulose-rich biomass [32].

Modifying the genes that regulate the activity of cellulose synthase complexes can also optimize cellulose production. Such modifications might, for instance, involve genes encoding proteins that enhance the formation and stability of the synthase complexes. Studies have shown that overexpression or knockdown of cellulose synthase genes, such as *PvCesA4* and *PvCesA6* in switchgrass (*Panicum virgatum*) [33], *AtCESA3(ixr1–2)* in *Nicotiana tabacum* L. [34], and *PmCesA2* in *Populus* sp. [35], can lead to altered cell wall composition, increased cellulose content, and improved growth performance.

Enhancing genes involved in the synthesis of UDP-glucose (the substrate for cellulose synthase) ensures an ample supply of building blocks for cellulose production. Overexpressed the UDP-glucose pyrophosphorylase gene in hybrid poplar (*Populus alba* x *grandidentata*) led to higher levels of UDP-glucose, which is directly used by cellulose synthase, and therefore the transgenic trees exhibited a significant increase in cellulose content and enhanced secondary xylem formation [36].

The overexpression of pectin methylesterase inhibitors (PMEIs) also can influence cellulose production by modulating the methylesterification status of pectin in plant cell walls. The suppression of modifying pectin methylesterase (PME) activity alters the cell wall’s pectin content, enhancing the cellulose quality and resulting in nanocellulose with improved characteristics [37].

### 2.3. Modified Cellulose Derivatives

Transgenic plants can also be modified to produce cellulose derivatives with altered properties, such as methylcellulose (MC), carboxymethyl cellulose (CMC), or hydroxypropyl cellulose (HPC). These modified cellulose derivatives have a very broad spectrum of applications in pharmaceuticals, food additives, and industrial products such as thickeners, stabilizers, and gelling agents.

Methylcellulose is one of the most important commercial cellulose ethers, widely utilized in various industrial applications. It is the most basic cellulose derivative, in which methyl groups (-CH3) replace the hydroxyl groups at the C-2, C-3, and/or C-6 positions of anhydro-D-glucose units. MC has amphiphilic properties alongside a set of other original physical and chemical qualities. Depending on the degree of substitution (DS), it is soluble in water or in organic solvents. Moreover, MC has the ability to undergo thermo-reversible gelation [38,39].

The MC production in transgenic plants involves techniques aimed at modifying plant cellulose to introduce methyl groups. Genes responsible for methylation need to be introduced into the plant’s genome; typically they encode methyltransferase enzymes that can add methyl groups to cellulose. These genes can be sourced from bacteria or fungi [40,41]. Furthermore, the cellulose biosynthetic pathway needs to be adjusted to accommodate methylation. Such adjustments might involve overexpressing native plant methyltransferases or inserting heterologous methyltransferase genes from other organisms [42].

Hydroxypropyl cellulose (HPC), like MC, can undergo thermo-reversible gelation in aqueous media. The sol-gel transition temperature of pure HPC is 44 °C, but different strategies can be used to achieve the hydrogel at lower temperatures for certain applications—for instance drug delivery systems require the transition temperature to be at 37 °C [43]. HPC production involves the introduction of hydroxypropyl groups into the cellulose backbone by partial etherification [44]. HPC is usually synthesized through a nucleophilic reaction between the hydroxyl groups of cellulose and electrophiles like alkyl halides or epoxides [45,46].

Carboxymethyl cellulose (CMC) is a water-soluble compound produced by chemically modifying cellulose through carboxymethylation. Valued for its high viscosity and low toxicity, it is also generally considered to be hypoallergenic [47]. In the food industry, the low viscosity type CMC acts as a moisture binder, and high viscosity type CMC acts as a gelation agent.

### 2.4. Cellulose-Based Fibers

Transgenic plants can be modified to produce cellulose fibers with tailored properties by inserting genetic modifications to enhance quality, increase yield, or introduce new functionalities. Modified cellulose-based fibers may have better strength, durability, or absorbency compared to conventional fibers, offering sustainable alternatives for various applications, for instance in textiles, paper, hygienic products, wound dressings, or bio-composites [48].

One of the most commonly genetically modified plants for the purpose of improving cellulose fiber qualities is cotton (*Gossypium* spp.). Tools such as transcriptomic studies and gene expression profiling provide insights into the genes and pathways suitable for genetic modifications towards enhanced fiber characteristics. The main focus is put on genes related to fiber development, such as those involved in hormone regulation, transcription factors, and cellulose synthesis. Specific genes (e.g., *MYB25*, *SUS*, *HOX1*, and *HD1*) involved in fiber initiation and growth have been identified and targeted for improvement [49,50].

Sucrose synthase (*SUS*) genes [49,51], MYB transcription factors (TFs) [52], and phytohormones, namely auxin [52,53], gibberellic acid (GA) [51,54], brassinosteroid (BR) [55], ethylene (ETH) [53], abscisic acid (ABA) [52], and cytokinin (CK) [52], are involved in the initiation mechanisms of lint and fuzz fibers. Modifications that affect these mechanisms aim to increase the number of fiber initials within the epidermal cells, thereby boosting fiber yield and its quality [50].

Overexpressing SUS genes in cotton plants has been shown to increase cellulose content and improve fiber quality by enhancing the supply of UDP-glucose for cellulose synthesis—for instance, the overexpression of the *GHSUSA1* gene in cotton led to increased fiber length and strength by promoting cell wall thickening during the development of the secondary cell wall. Additionally, high levels of *GHSUSA1* transcripts in vegetative tissues resulted in greater seedling biomass. In contrast, suppression of this gene reduced fiber quality and caused a decrease in yield as well as seed weight [56].

Several transcription factors, such as MYB, NAC, and WRKY, regulate the expression of genes involved in fiber development. The overexpression of the MYB transcription factor has been linked to improved fiber elongation and strength [50,57].

Targeting genes involved in the biosynthesis and signaling pathways of plant hormones, including manipulating the levels of certain hormones, can affect the initiation and elongation phases of fiber development [50]. Gibberellic acid (GA), auxin, ethylene (ETH), and brassinosteroid (BR) all contribute to fiber formation, while cytokinin (CK) and abscisic acid (ABA) inhibit fiber growth [58].

Higher GA levels are linked to the durability, micronaire, maturation, and elongation of cotton fibers [59]. Studies show that the levels of endogenous GA3 are significantly higher in cotton types with long fibers compared to those with medium and short fibers. The overexpression of GA20-OXIDASE1 (*GHGA20ox1*), a key enzyme in GA biosynthesis, results in increased fiber production and the production of longer fibers in transgenic cotton by significantly increasing the content of GA3 and GA4 [60]. Cotton fiber cells that overexpress GA20 oxidase exhibit significantly higher levels of *GHSUSA1* transcripts compared to wild-type cells. Additionally, the application of exogenous bioactive GA enhances *GHSUSA1* transcription in both fiber cells and hypocotyls [61].

In cotton, overexpression of the auxin synthesis gene, iaaM, with an ovule-specific promoter has been shown to increase the number of fiber initials [50].

Ethylene (ETH) is well known for regulating fruit ripening and organ abscission; however, it is also crucial in initiating and growing cotton fiber cells [62]. This is evidenced by the elevated expression of 1-aminocyclopropane-1-carboxylic acid oxidase (ACO1–3) genes during fiber elongation. Additionally, exogenous application of ETH has been shown to stimulate fiber cell elongation [63].

Brassinosteroids (BRs) are particularly important in the initiation and elongation of cotton fibers. In vitro studies show that low doses of BRs markedly enhance fiber cell elongation, while inhibiting BR production suppresses fiber cell growth [64]. The transgenic cotton plants overexpressing the BR-induced xyloglucan endotransglucosylase/hydrolase (XTH) gene *KC22* are able to produce significantly longer fibers [65].

### 2.5. Starch-Based Polymers

Starch is a widely distributed carbohydrate that can be found in various parts of plants such as leaves, seeds, fruits, stems, roots, and tubers. Structurally, starch consists of two types of polysaccharides: amylose, which consists of D-glucose residues linked by α-1,4 glycosidic linkages, and amylopectin, which contains α-1,4 glycosidic linkages with approximately 5% of α-1,6 glycosidic linkages. The presence of only α-1,4 glycosidic linkages is responsible for the linearity of amylose, while α-1,6 glycosidic linkages determine the branching of amylopectin. These components are combined within a water-insoluble granule that exhibits partial crystallinity. The size, shape, and overall structure of the starch granule as well as the amylose/amylopectin ratio depend on its biological origin [66].

Transgenic plants can be engineered to alter the natural proportion of the two polysaccharides, resulting in tailored starches with specific properties useful for various industrial applications, i.e., biodegradable plastics, pharmaceuticals, or food additives. Current research focuses on refining production processes, enhancing polymer properties, and addressing regulatory requirements to bolster the viability and acceptance of starch-based polymers derived from transgenic plants in commercial markets.

The commonly targeted genes are those involved in starch biosynthesis, such as granule-bound starch synthases (GBSS), soluble starch synthases (SSS), starch branching enzyme (SBE or BE), and debranching enzyme (DBE) [67,68]. Among these, SSS, BE, and DBE are specifically engaged in amylopectin synthesis, while GBSS is involved in amylose biosynthesis in various plant species, including cassava [69]. Silencing branching enzyme genes (BE1 and BE2) in transgenic cassava plants has induced significant changes in starch properties, increasing the amylose content up to [69].

Another approach is to explore the use of designer starch, such as amylose-only (AO) barley starch containing 99% amylose, for developing all-natural bioplastics that exhibit properties comparable to conventional plastic materials. This AO starch was created by RNA interference suppressing all three starch branching enzymes in barley (SBE1, SBE2a, and SBE2b) [70,71]. Prototypes of starch-based bioplastics, made from barley starch almost devoid of amylopectin synthesized within the grain itself, exhibited distinct properties when compared to the majority of high-amylose systems studied to date. The AO starches demonstrated significantly higher tensile stress at break and elongation at break compared to control barley starch extrudates, showcasing enhanced mechanical properties [71].

### 2.6. Polyhydroxyalkanoates (PHAs)

Polyhydroxyalkanoates (PHAs) are a large group of biopolymers naturally synthesized by numerous bacterial species [72]. However, their production is also possible in transgenic plants, presenting a promising alternative to the costly mass production of microorganisms. The first successful PHA production in transgenic plants was achieved in the 1990s when *A. thaliana* was transformed with genes encoding the enzymes needed for polyhydroxybutyrate (PHB) synthesis, which were isolated from *Alcaligenes eutrophus* [11].

PHA are polymers composed of 600 to 35,000 identical monomers. These polymers share similar properties with widely used petroleum-based plastics, but they are biodegradable, making them an excellent alternative to synthetic materials that take a long time to decompose. The physical properties of PHAs primarily depend on the type of monomers they are constructed from, for example being insoluble and impermeable to air. Other homopolymers mostly exhibit similar characteristics to PHB. However, copolymers containing PHB are stronger, less rigid, less brittle, and much more flexible than PHB homopolymers. Compared to short-chain-length PHAs (scl-PHA), PHAs made from medium-length monomers (mcl-PHA) are less crystalline and more elastic [73]. The significant differences in the physical and chemical properties between scl-PHA and mcl-PHA drive the search for a polymer with intermediate features. Copolymers containing both scl-PHA and mcl-PHA meet these conditions. However, biosynthesizing such structures in plant cells is challenging due to the need to control the appropriate ratio and composition of the monomers involved [74].

The synthesis of PHAs in transgenic plant cells is feasible due to the widespread availability of acetyl-CoA, a key substrate in PHA biosynthesis. Cellular compartments particularly rich in acetyl-CoA include the cytoplasm, plastids, peroxisomes, and mitochondria, and have been utilized in attempts to synthesize and accumulate various types of PHAs. However, mitochondria have proven to be the least effective for this purpose, mainly due to the rapid consumption of acetyl-CoA in cellular respiration [75].

Some of the most significant research has been conducted on the well-known model plant, *A. thaliana*. This species has achieved the highest level of biopolymer accumulation in the form of PHB to date. However, *A. thaliana* is not a crop plant suitable for large-scale use as a bioreactor for PHA production. With the extensive knowledge available on plant genetic modifications and the biosynthetic pathways of various PHAs, we are now focusing on plants with increased acetyl-CoA metabolism. A notable success was the modification of *Camelina sativa*, achieving a PHB accumulation of 15% in the seeds of the first generation [76]. Challenges remain in increasing PHA accumulation efficiency and improving the health of tissues where polymers are stored.

### 2.7. Spider Silk Proteins

Fibrous proteins of spiders and insects are large molecules, often several hundred kDa, and exhibit highly repetitive amino acid sequences. The genes encoding several spider silk proteins and insect fibroins from different species have been characterized, providing insights into their evolutionary implications. These proteins are characterized by a modular nature, with repetitive portions that can be generalized into typical sets of consensus repeats. In spiders, characterized silk proteins contain six types of amino acid motifs: poly-A, poly-(GA), GGX, GPGXX, GPX, and spacers [77]. These repetitive motifs are believed to be directly responsible for the mechanical properties of spider silk fibers. Poly-A and poly-(GA) sequences are found in the β-sheet regions of major and minor ampullate silks. These β-sheet regions are capable of linking to form crystalline areas within the fibers, which contribute to the extremely high tensile strength of silk fibers. Additionally, the GPGXX motif is also thought to enhance the mechanical properties of silks [78].

When expressed in microorganisms such as *E. coli* or in lower eukaryotes like *Pichia pastoris*, transgenic spider silk-like proteins accumulated only at low levels. Due to the high content of hydrophobic amino acids, glycine, and alanine in spider silk proteins, a substantial supply of these building blocks is required for their production in fast-growing microorganisms like bacteria or yeast [79,80]. Alternatively, fragments of the spider silk proteins MaSpI, MaSpII, and Adf3, ranging from 60 to 140 kDa, have been produced in cultured mammalian cells. However, mass production from animal cells or transgenic animals remains too costly and time-consuming due to fermentation or breeding requirements [81]. Transgenic plants present a promising solution to these production challenges. They can act as efficient protein factories, offering a feasible alternative to microbial and animal systems for the large-scale production of spider silk proteins [82].

Synthetic silk genes have been successfully expressed in transgenic tobacco, potato, and *A. thaliana*. In a 2001 study [83], the synthesis of high molecular weight spider silk proteins in plants was successfully demonstrated.

### 2.8. Rubber

Natural rubber, a highly significant biological material used in various non-food applications, is a polymer composed of isoprene units connected in a 1,4-cis configuration. Rubber’s properties include resilience, elasticity, abrasion, and impact resistance, efficient heat dispersion, and malleability at low temperatures [84,85]. These unique properties of natural rubber make it essentially irreplaceable for many applications, such as heavy-duty tires for trucks, buses, and airplanes, as well as latex products for medical applications [84].

Rubber biosynthesis (RB) occurs on the surface of a special type of organelle (rubber particle) in the cytoplasm (latex) of the rubber-producing laticifers or latex vessels [86]. In *Hevea brasiliensis*, the general metabolic pathway for rubber biosynthesis is now well understood, with all the involved genes identified, largely due to the use of Illumina second-generation sequencing technology on *Hevea brasiliensis* latex and bark [87,88,89]. Key RB genes, such as cis-prenyltransferase (CPT) [90], rubber elongation factor (REF) [91], small rubber particle protein (SRPP) [92], and hydroxymethyl glutaryl coenzyme A reductase (HMGR) [93], have been cloned and thoroughly characterized. However, the expression of genes in other metabolic pathways within latex cells is crucial for latex regeneration, particularly during ethylene-stimulated latex production. Overall, our understanding of the molecular regulation of rubber productivity remains limited.

Although more than 2500 plant species naturally synthesize rubber, most have unfavorable characteristics, such as very low rubber yield or low molecular weight of the polymer [94]. *H. brasiliensis*, commonly known as the rubber tree, is almost the exclusive species producing commercially viable natural rubber [95]. However, this tree has specialized growth requirements and is susceptible to fungal infections, and its harvest is labor-intensive. As a result, new rubber-producing species, such as Russian dandelion (*Taraxacum koksaghyz*), guayule (*Parthenium argentatum*), and hardy rubber tree (*Eucommia ulmoides*), are being domesticated for industrial rubber production in colder and less productive geographical areas [94,96].

The Russian dandelion (*T. koksaghyz*) produces high molecular mass cis-1,4-polyisoprene in specialized latex-producing cells called laticifers making it a potential alternative source of natural rubber [97]. While laticifers are also present in pedicels and leaves, natural rubber is primarily synthesized in the root system. In a 2016 study, the CRISPR/Cas9-induced genome editing technique was rapidly introduced into *T. koksaghyz* hairy roots using *Agrobacterium rhizogenes*, successfully regenerating whole plants with edited genomes within just six weeks. The target gene for editing was fructan: fructan 1-fructosyltransferase (1-FFT), due to its role in inulin biosynthesis, which antagonizes rubber production. The idea was to minimize metabolites that compete with rubber for energy, consequently enhancing rubber production in the plant. Mutagenesis was confirmed by the loss of restriction sites within 1-FFT and subsequent sequencing, with mutated plants showing a mutation rate as high as 80.0%. These findings indicate a significant potential for increasing rubber yield in *T. koksaghyz* through targeted genome editing using CRISPR/Cas9 and *A. rhizogenes* [98].

## 3. Transgenic Plants in Artificial Biopolymer Production

Both transgenic plants and microorganisms have been utilized for artificial biopolymer production, each presenting distinct advantages and challenges. Transgenic plants capitalize on renewable resources like sunlight, water, and carbon dioxide for growth, reducing reliance on finite fossil fuel reserves and minimizing environmental impact. They can be cultivated using conventional agricultural practices, resulting in relatively low production costs [99]. In contrast, microbial bioreactors require expensive media and highly controlled growth conditions, making them less advantageous in terms of scalability and economic factors. Plant molecular farming is poised for a bright future due to its ability to produce a wide array of recombinant molecules with desirable post-translational modifications, which are often unattainable in bacterial production systems. This approach offers significant flexibility for engineering a variety of products, including artificial biopolymers and biodegradable plastics, as well as pharmaceutical proteins [99,100,101].

Despite their advantages, transgenic plants face several challenges. Development times are long, regulatory hurdles can be significant, and there are potential ecological impacts to consider. Public perception and the general lack of acceptance of genetically modified organisms can also impact the marketability of artificial biopolymers produced in transgenic plants. Moreover, achieving high expression levels of these molecules in plants is often difficult. Many transgenic plants produce low yields of the desired polymer, necessitating further modifications and techniques to increase yield. Nonetheless, their potential for large-scale, cost-effective, and sustainable artificial biopolymer production makes them a promising alternative to microbial bioreactors.

### Plant Species Selection

Selecting the appropriate host plant species is crucial for the efficient production of recombinant products. While tobacco, known for its high susceptibility for transformation and manipulation, was traditionally the preferred system for producing plant-derived molecules, many other plant species are now being utilized. These include tomato, banana, rice, maize, wheat, carrot, soybean, pea, potato, lettuce, and alfalfa. Each species has specific requirements for production, making it clear that no single species can be ideal for producing all types of recombinant products. Domesticated species are generally preferred over wild species for molecular farming due to their adaptation to a wide range of environmental conditions. However, wild species could offer advantages by preventing confusion with food crops, thus addressing concerns about transgenic material mixing with food crops. Self-pollinating species are also beneficial for containment purposes, as they reduce the risk of transgene spread through pollen [99,102].

Tobacco offers several unique advantages as a production platform. As a non-food crop native to South America, it has no close relatives in Europe and North America, and it does not persist in northern climates, eliminating the risk of the occurrence of mix-ups out-crossing [95]. These traits, coupled with its high biomass yield, ease of genetic transformation, and ability to utilize plastid transformation for efficient gene expression, position tobacco as a promising bioreactor—it has already been modified to produce artificial biopolymers including PHAs and spider silk proteins. However, the initial PHB production in tobacco, using a plastidial promoter and 5′UTR preceding the native bacterial operon, resulted in only up to 1.7% PHB of dry weight in tobacco leaf plantlets regenerated from callus [103]. Therefore, in a 2011 study [104], plastid-encoded PHB production in tobacco was revisited to investigate the potential for stable, high-level polymer production in soil-grown plants and to explore plastid-encoded gene expression as a reliable system for engineering multigene pathways to produce industrial products. Efforts focused on enhancing and stabilizing transgene expression by extending the photosynthetic related *PSBA* operon with four transgenes: the three PHB pathway genes and a selectable marker. Short translational control elements were employed to optimize expression of each transgene. Additionally, for further improvement of expression, genes with similar codon usage and GC content to the native tobacco plastome were selected. This approach enabled the production of significantly higher levels of PHB in both heterotrophic and autotrophic plants compared to previous studies. In greenhouse-grown plants, average PHB levels of 10% to 18% of dry weight were observed in leaf tissue samples from transgenic lines [105].

In addition to tobacco, other leafy crops such as lettuce, alfalfa, and *A. thaliana* are also used as bioreactors. Alfalfa stands out for several reasons—it is a perennial plant that can fix its own nitrogen, and the glycoproteins produced in its leaves typically have homogeneous glycan structures. It also is exceptional at adapting to a wide range of environments. Due to its qualities, alfalfa as a bioreactor for production of raw materials, including PHB, offer lower production costs of artificial biopolymers, as multiple harvests could occur each year with little fertilization or management costs. In a 2002 study [106], three genes from *Ralstonia eutropha* encoding enzymes crucial for PHB synthesis—namely PHBA, PHBB and PHBC—were introduced into alfalfa plants through *Agrobacterium*-mediated transformation. The yield levels observed in transgenic alfalfa plants were lower compared to those reported in other model plants, necessitating an increase to make production economically feasible.

*A. thaliana* is another example of a plant system that was extensively experimented on to obtain high levels of artificial biopolymer accumulation within its tissues. Production of poly-β-hydroxybutyrate (PHB) in *A. thaliana* leaves was first demonstrated using *Ralstoniaeutropha* enzymes, achieving polymer accumulation of up to 14% of the plant’s dry weight when the PHB biosynthetic enzymes were targeted to the chloroplast [107].

In contrast, proteins expressed in cereal grains are shielded from proteolytic digestion, allowing them to remain stable for extended periods at room temperature without significant loss of activity. Various cereals, such as rice, wheat, barley, and maize, have been studied for this purpose.

Notably, maize was used to produce the first commercially available plant-derived product. The transgenic maize seeds contained avidin, a glycoprotein originally found in the egg whites of birds, reptiles, and amphibians [108]. It has been shown that commercial production of avidin in plants is both viable and advantageous compared to the traditional method of extracting the protein from chicken egg white. Plant-based production benefited from lower raw material costs and required significantly less starting material. Additionally, the protein remained stable in maize seeds and retained reasonable stability during processing. These factors contributed to a reduced cost of producing avidin commercially, enhancing the credibility of this production system. This marked the first instance of using plants to produce a heterologous protein for commercial purposes [108].

Barely was used to explore the production of amylose-only (AO) starch with an amylose content of 99%. This specialized starch was achieved by using RNA interference to suppress all three starch branching enzymes [70,71]. Prototypes of starch-based bioplastics, derived from barley starch nearly lacking amylopectin naturally synthesized within the grain, demonstrated unique properties compared to the predominant high-amylose systems studied previously, showcasing the potential of such techniques for artificial biopolymer production.

Conversely, oil crops offer unique production advantages, particularly in terms of cost-effective downstream processing when proteins are targeted to oil bodies. Oleosin, a plant protein, is located on the surface of oil bodies. Its hydrophobic central core remains embedded within the oil body, while the amphipathic and less conserved N- and C-terminals are exposed on the surface. Proteins can be targeted to oil bodies as oleosin fusions, which can later be isolated from most contaminants through centrifugation-based methods. This approach simplifies the purification process, making it more efficient and cost-effective [99].

*Brassica napus* oilseed was seen as a promising alternative system for PHB production, as acetyl-CoA, the substrate necessary for the initial step of PHB biosynthesis, is abundantly available during fatty acid synthesis [109]. Therefore, in 1999, *B. napus* plants were transformed with a single multi-gene vector sequentially assembled from gene cassettes containing genes from the bacterium *R. eutropha* encoding a β-ketohexose, an acetoacetyl-CoA reductase, and a PHB synthase—three key enzymes for PHB synthesis. As a result, PHB accumulated in the leukoplasts of mature seeds, reaching levels as high as 7.7% of the fresh seed weight [109].

## 4. Applications of Artificial Biopolymers

**Figure 1:**
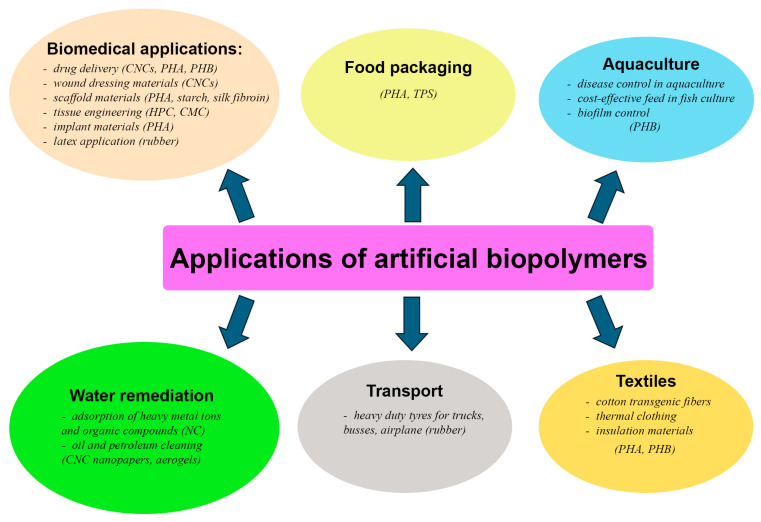
Areas of application of artificial biopolymers derived from transgenic plants. CNCs—cellulose nanocrystals, PHA—polyhydroxyalkanoates, PHB—polyhydroxybutyrate, HPC—hydroxypropylcellulose, CMC—carboxymethyl cellulose, TPS—thermoplastic starch, NC—nanocellulose.

Environmental pollution from conventional polymers and its impact on practically all ecosystems on Earth is a major driving force behind the development of biodegradable and sustainable polymers. Alongside this, the growing ecological awareness among the population highlights the need to implement artificial biopolymers as alternatives to petroleum-derived polymers. Artificial biopolymers have garnered significant attention for use in applications requiring sustainable and biodegradable products. These materials are expected to help mitigate environmental pollution and reduce municipal waste caused by non-biodegradable products, particularly if they are soil degradable. Artificial biopolymers find diverse applications (Figure 1), ranging from simply serving as eco-friendly alternatives to petroleum-derived plastics to pioneering advancements in pharmaceutical technologies and water remediation techniques.

### 4.1. Biomedical Applications

In recent years, there has been growing interest in the use of natural polymers in biomedical applications. Currently, a significant portion of the waste generated by the healthcare industry consists of single-use plastics, which are gradually being replaced by more sustainable alternatives. However, artificial biopolymers offer more than just a direct substitute for petroleum-based plastics in packaging and sanitary products. They also demonstrate immense potential for applications in innovative areas such as drug delivery systems, wound dressings, and tissue engineering (Table 1).

Encapsulating drugs with coating materials is highly recommended to achieve sustainable, controlled release over a specific period. Generally, drug release relies on the principle of diffusion and involves three successive steps: absorption of the liquid from the gastrointestinal environment, swelling of the active elements in the drug, and the subsequent collapse of the drug structure [8]. Among the various types of nanofillers, cellulose nanocrystals (CNCs) have garnered significant interest for biomedical applications due to their exceptional biocompatibility, renewability, and high elasticity. Because of their high specific surface area, colloidal stability, negative charge density, and excellent mechanical properties, cellulose nanocrystals (CNCs) hold immense potential as effective pharmaceutical excipients and carriers in drug delivery systems. The unique properties of CNCs enable the loading of both charged and neutral drugs, controlled release of active compounds, and targeted delivery to specific cells [110,111]. Moreover, CNCs’ hydrophilic nature and chiral nematic structure, which fosters porous formations, make them ideal drug excipients, especially in hydrogel formulations. Integration of CNCs into porous hydrogels enhances drug absorption and dissolution processes due to their strong hydrophilic properties and water-swelling behavior. The high permeability of CNC hydrogels has made them widely utilized in studies converting chemical, pH, thermal, and electrical signals into conformational changes that trigger drug release, with pH manipulation being particularly prominent [112]. However, the hydrophilic nature and highly negative surface properties of CNCs pose limitations on their application for delivering hydrophobic drugs, such as certain anticancer medications. Thus, additional surface modification treatments of CNC can alter their properties to effectively bind non-ionized or hydrophobic drugs that would not typically interact with untreated CNC [8].

Wound dressing materials are engineered to support wound healing by creating a barrier against bacterial penetration, promoting gas exchange, treating infections, and facilitating wound recovery. In recent decades, there has been increasing interest in utilizing naturally occurring polymers and their hybrids in wound dressing materials. These materials are valued for their biocompatibility, biodegradability, and low cytotoxicity. Among various natural polymers, cellulose nanocrystals (CNCs) have emerged as useful components in wound dressings, either as reinforcing agents [113] or as primary building blocks, owing to their widespread availability and excellent mechanical properties [114]. Wound dressing materials made from cellulose nanocrystals (CNCs) can be developed as films, hydrogel pads, or injectable hydrogels. These CNC-based wound dressings can be enhanced by incorporating active compounds such as antimicrobial agents, antioxidants, hormones, enzymes, and vitamins. This modification aims to impart self-healing properties to the CNC-based wound dressing system [8].

One of the simplest acellular approaches in regenerative medicine involves implanting a scaffold device designed to actively enhance the body’s natural healing and self-repair processes. These scaffold materials must mimic the function of the extracellular matrix (ECM) to promote cellular invasion, attachment, and proliferation, thereby facilitating tissue regeneration. In the current standard approach to tissue engineering, cells (especially stem cells, whether autologous or allogeneic) are cultured on biodegradable and bioactive scaffolds that replicate the ECM. This guides their attachment, migration, proliferation, and differentiation to produce 3D tissue substitutes intended for implantation [115]. Polysaccharides like starch, proteins such as silk fibroin, natural biopolyesters like polyhydroxyalkanoates (PHA), and a variety of biofibers such as lignocellulosic fibers have been utilized in scaffold design [116,117]. Cellulose nanocrystals and several chemically modified cellulose derivatives, including hydroxypropylcellulose (HPC) [118] and carboxymethyl cellulose (CMC), have also found applications in tissue engineering. CNCs exhibit low toxicity, low density, high aspect ratio, and importantly, promising mechanical properties, making them ideal for constructing functional tissue substitutes, which are crucial for repairing damaged tissues or organs [117].

Polyhydroxyalkanoates (PHAs) have been extensively used as implant materials in various applications within the human body, including sutures, screws, staples, bone plates, stents, adhesion barriers, devices for articular cartilage repair, and nerve guides. Their properties, such as biodegradability, biocompatibility, and the ability to stimulate bone growth and wound healing, make PHAs a promising candidate for numerous biomedical applications, including implants. Additionally, the mechanical properties of PHAs, such as their elasticity modulus and tensile strength, further enhance their potential as biomedical implants. Moreover, PHA-based implants maintain a stable pH during degradation, ensuring compatibility with the host immune system [119].

### 4.2. Food Packaging

Food packaging plays a crucial role in ensuring food safety by serving as a protective barrier shielding food from physical, chemical, and biological hazards. The artificial biopolymers most frequently utilized in food packaging include PHAs, thermoplastic starch (TPS), cellulose, and proteins (Table 1). These are preferred because of their widespread availability, lack of odor, and non-toxic nature. To qualify as suitable food packaging materials, artificial biopolymers must satisfy specific criteria. These include safeguarding food quality from contamination, ensuring non-toxicity upon contact with food, providing effective barrier properties, and possessing sufficient mechanical strength [13]. Cellulose, known for its high thermal resistance, can also serve as a protective barrier against ultraviolet rays. Additionally, cellulose possesses the capability to transport antioxidant and antibacterial agents. Cellulose-based fibers are valuable materials due to their essential properties—they are biodegradable and renewable, making them suitable for various polymer composite applications. Moreover, they are non-corrosive, combustible, and non-toxic [22].

Polymer materials commonly used in packaging often do not provide complete barriers against small molecules like water vapor, gases, and organic substances, which can negatively impact their effectiveness and suitability for food packaging applications. Several strategies have been explored to address these limitations. One prominent approach to bolster polymer barrier properties is by integrating CNC as both reinforcing agents and permeability enhancers. CNC has proven to be versatile in improving the performance of polymer materials, not only in creating high-performance nanocomposite films but also in serving as a moisture-absorber for packaged foods [8].

### 4.3. Water Remediation

Industries often release significant impurities, including heavy metals, dyes, pesticides, polycyclic aromatic hydrocarbons, chemicals, and biomolecular contaminants, which can enter water sources through various channels. Nanocellulose, with its intrinsic properties, offers enhanced performance in water treatment. Its high surface area, tensile strength, ability to modulate surface chemistry through anionic or cationic grafting, and hydrophilicity make it particularly valuable for wastewater treatment. Functionalized nanocellulose, along with nanocomposites, nanohybrids, and superhydrophobic membranes, have proven to be effective adsorbents for a range of pollutants, i.e., organic contaminants from drainage discharge, heavy metal ions, oil, dyes from industrial wastewater, and pathogens in contaminated water [120]. Ultra-porous nanocellulose aerogels are employed to separate water and petroleum mixtures, while CNC nanopapers and nano-coatings are effective in cleaning up crude oil spills in saltwater, providing a promising solution to water pollution [121].

### 4.4. Aquaculture

The use of PHA for disease control in aquaculture has shown outstanding results. Research on *Vibrio campbelli* and *Artemia franciscana* demonstrated the inhibitory and anti-adhesive properties of PHB [122,123]. Due to the surface accumulation of methyl groups, PHB has less cell adhesion and disrupts the formation of biofilms, diminishing biomass formation. Thus, it controls pathogen growth. Other studies have highlighted PHB’s anti-adhesive effects against shrimp pathogens like *Vibrio alginolyticus*, *Vibrio harveyi*, *Vibrio fischeri, Vibrio vulnificus*, and *Vibrio parahaemolyticus*. PHB also has been shown to increase larvae survival rates after infection with *Edwardsiella ictaluri* [124]. Beyond its anti-infective capabilities, PHB can serve as a cost-effective feed in fish culture, providing an excellent source of energy [119].

**Table 1:** Applications and biological origins of different types of artificial biopolymers derived from transgenic plants.

Type of Artificial Biopolymer	Transgenic Plant Used as a Source	Application	References
Cellulose-based polymers	*Panicum virgatum*, *Nicotiana tabacum*, *Populus* sp.	wound dressing materials, drug delivery systems, medicinal inserts, implants, tissue engineering, cosmetic agents, food packaging, water remediation	[25,33,34,35,110,111,120,121]
Starch-based polymers	*Hordeum* L.	biodegradable plastics, pharmaceuticals, food packaging	[69,70,71]
Polyhydroxyalkanoates	*Arabidopsis thaliana*, *Camelia sativa*, *Nicotiana tabacum*, *Medicago sativa*, *Brassica napus*	implant materials (including sutures, screws, staples, bone plates, stents), food packaging	[11,13,76,119]
Spider silk proteins	*Nicotiana tabacum*, *Solanum tuberosum* L. *Arabidopsis thaliana*	textiles, biomedical materials, silk films, tissue engineering	[125,126]
Rubber	*Hevea brasiliensis*, *Taraxacum koksaghyz*, *Parthenium argentatum*, *Eucommia ulmoides*	heavy-duty tires (trucks, buses, airplanes), latex products for medical applications	[84,94,95,96]

## 5. Limitations

Artificial biopolymers have a number of advantageous features compared to conventional, petroleum-based synthetic materials. They are biodegradable, renewable, have low or zero toxicity, and are carbon neutral. Petroleum-based materials, in turn, are often indicated as causes of allergies, cancers, and other serious diseases, especially in young children and pregnant women [127]. Their production is associated with high CO_2_ emissions into the atmosphere.

Although artificial biopolymers are seen as a promising solution to mitigate the environmental pollution caused by traditional polymers, it is crucial to develop a comprehensive and nuanced understanding of their suitability for specific applications. For example, biodegradable polymers should not be applied where the long-term stability and resistance to environmental factors are the most important consideration. Additionally, minimizing the costs of artificial biopolymers remains crucial to widen their areas of application and meet the required cost-performance ratios. For example, one of the main drawbacks in the use of CNCs in many commercial applications is related to their efficient production at affordable quantity and quality. However, the initial demonstration plants for the commercial-scale production of CNCs are now being developed in Canada and the U.S.A. It is anticipated that mass production will soon increase to multiple tons per day, significantly enhancing their application in industrial-scale composites [117].

Recycling of artificial biopolymers, unlike that of conventional polymers, is not yet well-established. The low production volumes of artificial biopolymers pose a challenge to their recyclability. It has been estimated that global production of artificial biopolymers needs to reach at least 200 kilotons, with a recycling capacity of 5–18 kilotons annually, for a recycling plant to be profitable [128]. This raises questions about the actual environmental impact of artificial biopolymers. However, there is significant patenting activity related to biopolymer recycling, offering hope for future solutions. Separate recycling streams are expected to develop as the production and utilization of artificial biopolymers increase. Composting is a viable alternative to landfill disposal for most biodegradable molecules. The compostability of artificial biopolymers can be enhanced by blending them with other polymers or fillers. Soil contains various microorganisms (bacteria and fungi) that can use biopolymers as an energy source, and numerous studies have demonstrated the feasibility of biodegradation of biopolymers in soil burial [13,129,130,131]. However, this applies only to some of the materials classified as biopolymers. It should be understood that many artificial biopolymers cannot be recycled, at least with our current knowledge.

## 6. Conclusions

The production of artificial biopolymers in transgenic plants presents a promising avenue for sustainable materials, leveraging plants as biofactories for compounds such as nanocellulose, polyhydroxyalkanoates, starch-based polymers, or natural rubber. Future developments in this field are likely to focus on enhancing the yield and quality of these molecules, optimizing genetic modifications for higher efficiency, and scaling up production processes to meet industrial demands. Despite their potential, artificial biopolymers face several limitations including their unsuitability for applications requiring long-term stability and resistance, as well as relatively high production costs and low volumes that pose challenges for commercial use and waste management. While efficient production methods are being developed, they are not yet widespread, and recycling systems for artificial biopolymers remain underdeveloped, necessitating substantial production volumes to be economically viable. Nonetheless, advancements in composting and soil biodegradation may offer alternative disposal methods. Integrating artificial biopolymers into existing recycling and composting frameworks will be crucial for maximizing their environmental benefits. Progress in synthetic biology and plant biotechnology will lead to more robust and cost-effective production systems, ultimately broadening the applications of plant-derived artificial biopolymers and contributing significantly to reducing reliance on fossil-based plastics.
